# New York School of Clinical Medicine

**Published:** 1900-08

**Authors:** Marcus Kenyon

**Affiliations:** Secretary New York School of Clinical Medicine. New York


### NEW YORK SCHOOL OF CLINICAL MEDICINE.

*To the Editor, Buffalo Medical Journal:*

SIR.—The New York School of Clinical Medicine has not been discontinued. Will you kindly publish this statement in order to refute the erroneous announcement to the contrary appearing in a few of the medical journals?

Very truly yours,

MARCUS KENYON, M. D.,

*Secretary New York School of Clinical Medicine.*

NEW YORK, July 12, 1900.

